# *In silico-designed* mutations increase variable new-antigen receptor single-domain antibodies for VEGF_165_ neutralization

**DOI:** 10.18632/oncotarget.25549

**Published:** 2018-06-15

**Authors:** Dalia Millán-Gómez, Salvador Dueñas, Patricia L. A. Muñoz, Tanya Camacho-Villegas, Carolina Elosua, Olivia Cabanillas-Bernal, Teresa Escalante, Almudena Perona, David Abia, Florian Drescher, Pierrick G. J. Fournier, Marco A. Ramos, Rosa E. Mares, Jorge Paniagua-Solis, Teresa Mata-Gonzalez, Jorge Gonzalez-Canudas, Robert M. Hoffman, Alexei Licea-Navarro, Noemí Sánchez-Campos

**Affiliations:** ^1^ Departamento de Innovación Biomédica, Centro de Investigación Científica y de Educación Superior de Ensenada (CICESE), Ensenada, Baja California, México; ^2^ Facultad de Ciencias Químicas e Ingeniería, Universidad Autónoma de Baja California, Tijuana, Baja California, México; ^3^ CONACYT- Unidad de Biotecnología Médica y Farmacéutica, Centro de Investigación y Asistencia en Tecnología y Diseño Del Estado de Jalisco (CIATEJ), Guadalajara, Jalisco, México; ^4^ Teraclon IDF, Parque Científico de Madrid, Tres Cantos, Madrid, Spain; ^5^ Instituto Clodomiro Picado, Facultad de Microbiología, Universidad de Costa Rica, San José, Costa Rica; ^6^ Grupo de Biotecnología Aplicada, Universidad Europea de Madrid, Madrid, Spain; ^7^ Laboratorios Silanes S.A. de C.V., Ciudad de México, México; ^8^ AntiCancer Inc., San Diego, CA, USA; ^9^ Department of Surgery, University of California, San Diego, CA, USA

**Keywords:** VNAR, shark antibody, VEGF neutralization, in silico mutation, angiogenesis

## Abstract

The stability, binding, and tissue penetration of variable new-antigen receptor (VNAR) single-domain antibodies have been tested as part of an investigation into their ability to serve as novel therapeutics. V13 is a VNAR that recognizes vascular endothelial growth factor 165 (VEGF_165_). In the present study V13 was used as a parental molecule into which we introduced mutations designed *in silico*. Two of the designed VNAR mutants were expressed, and their ability to recognize VEGF_165_ was assessed *in vitro* and *in vivo*. One mutation (Pro98Tyr) was designed to increase VEGF_165_ recognition, while the other (Arg97Ala) was designed to inhibit VEGF_165_ binding. Compared to parental V13, the Pro98Tyr mutant showed enhanced VEGF_165_ recognition and neutralization, as indicated by inhibition of angiogenesis and tumor growth. This molecule thus appears to have therapeutic potential for neutralizing VEGF_165_ in cancer treatment.

## INTRODUCTION

As single-domain antibodies, variable new-antigen receptors (VNARs) are attractive as potential therapeutics due to their small size, which reduces recognition by the immune system, as well as their increased solubility, thermal stability, and ability to refold after denaturation [1–4]. VNARs have a high-variability CDR3, which acquires an extended hairpin shape that allows insertion into cryptic epitopes [5–8]. Even when VNARs have sub-nM affinities [9], it is possible to improve their antigen binding through various techniques, including phage display, random mutagenesis, site-directed mutagenesis, and *in vitro* maturation affinity [10, 11]. However, these techniques entail replacing amino acids, whether or not they are in contact with the antigen. *In silico* analysis enables one to model interactions between amino acids and construct docking sites in which it is possible to determine regions with more or less free energy [12].

**Figure 1:**
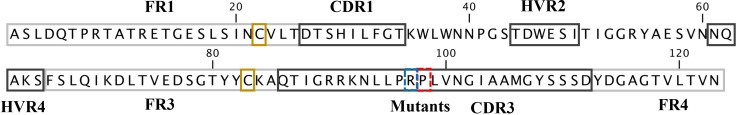
Amino acid sequence of VNAR V13 Single letter codes are used for the V13 sequence, yellow boxes show canonical Cys, the blue box represents mutated Arg97, which decreased VEGF_165_ recognition, and red box represents mutated Pro98, which increased VEGF_165_ recognition.

VEGF_165_ is an angiogenic cytokine that also regulates vascular permeability and promotes migration of endothelial cells [13–15]. Angiogenesis is critical for the early development of cancer and for metastatic spread [16]. There are two monoclonal antibodies against VEGF_165_ on the market, only one of which is used for cancer therapy [17]. One possible adverse reaction to this antibody is hypersensitivity, causing anaphylaxis and bronchospasm. Using a previously-isolated and well-characterized anti-VEGF_165_ VNAR (Figure 1) [18], we performed an *in silico* mutation analysis to identify amino acids that provide greater interaction energy so as to increase VEGF_165_ recognition and improve its neutralization with a novel *in silico*-designed VNAR.

## RESULTS

### Refinement of VNAR V13 modeling with MD

Figure 1 shows the amino acid sequence of V13 and the most important regions from a VNAR. After modeling by threading, the V13 model was refined using MD in which five possible conformations were generated, differing primarily with regard to CDR3. The conformation that had a longest time of existence was selected. Theoretically, this conformation should have the most stable structure (Conformation 4 in Supplementary Table 1).

### Models of VNAR V13 -VEGF_165_ docking

Four different models (A-D) of interaction between V13 and VEGF_165_ were generated (Supplementary Figures 1, 2). To select the models that best explain the optimal binding between VEGF_165_ and V13, a comparative analysis of the interaction energy of each model was performed (Table 1). In Model A, five strong interactions were identified (hydrogen and ionic bonds) between the CDR3 recognition loop of V13 and different amino acids in chain A or B of VEGF_165_ [V13-Arg97 with VEGF_165_(B)-Glu51 and VEGF_165_ (A)-Asp21]. Also identified were additional stabilizing interactions [V13-Arg90 with VEGF_165_ (B)-Glu60, V13-Arg91 with VEGF_165_ (B)-Glu25, and V13-Lys92 with VEGF_165_ (B)-Glu54].

**Table 1:** Interaction energy of the models chosen for the VEGF_165_-V13 complex

Models	E_total_	E_vdw_	E_HB_	SASA	E_total_/SASA	E_vdw_/SASA	E_HB_/SASA
Control	-144.04	-101.30	-19.42	893.50	0.1612	0.1134	0.1351
Model A	-113.53	-60.13	-16.73	809.10	0.140	0.074	0.095
Model B	-112.41	-92.92	-11.19	549.50	0.204	0.169	0.189
Model C	-131.10	-81.53	-14.91	870.40	0.151	0.094	0.111
Model D	-109.57	-90.99	-10.27	1000.60	0.110	0.091	0.101

The energy and occluded surface values were calculated for the minimal average structure in the last 500 ps of the dynamics.

E_total_: total energy; E_vdw_: Van der Waals energy; E_HB_: energy hydrogen bonds; SASA: solvent accessible surface area.

In Model B, the interactions were not as robust as in Model A, but they were distributed more widely throughout V13. We observed weak interactions between amino acids in the CDR3 recognition loop and VEGF_165_ chains A and B.

Model C had the same pattern of interactions as Model A, but the interaction energy was higher than in model A. This is represented by bonds between the CDR3 recognition loop and VEGF_165_ chain A [V13-Arg97 with VEGF_165_ (A)- Glu54 and V13-Asn101 with VEGF_165_ (A)-Lys94] and additional stabilizing interactions [V13-Arg90 with VEGF_165_ (A)-Glu60, V13-Arg91 with VEGF_165_ (A)-Glu25, V13-Lys92 with VEGF_165_ (A)-Glu54 and VEGF_165_ (A)-Cys55 and V13-Tyr108 with VEGF_165_ (A)-Glu25].

In Model D, the interactions in Models A and C were recapitulated but weakly. Model C was therefore selected as representing the complex of VEGF_165_ with V13, as it had better total energy values at the end of the dynamics and the optimal interaction pattern.

### Structural and energetic analysis of two VNAR V13 mutants in complex with VEGF_165_

**Figure 2:**
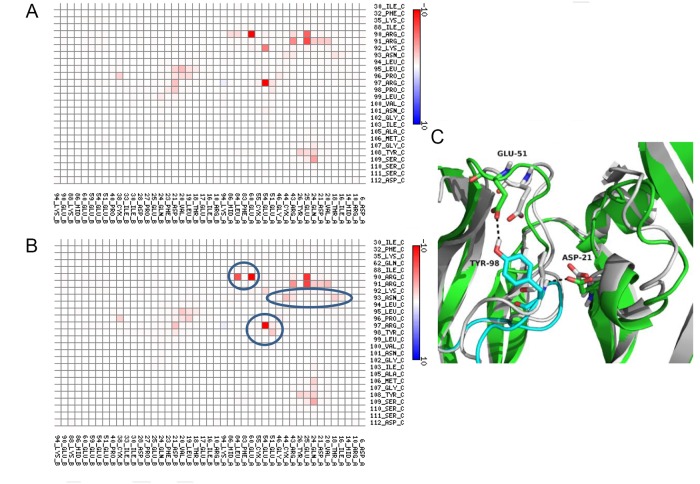
Map of interactions of mutant 1 (Pro98Tyr) within the VNAR V13-VEGF_165_ complex Interactions between V13 (represented on the vertical axis) and its VEGF_165_ receptor (represented of the horizontal axis) are shown for complexes with the parental **(A)** and mutated **(B)** species. The color scale is a function of the interaction energy: redder shades indicate more favorable interactions, while bluer tints are less favorable. **(C)** Representation of the interaction at position 98 of V13 and position 51 of VEGF_165_ before (white cartoon) and after (VEGF_165_ green cartoon and V13 cyan cartoon) mutation. Blue circles show the amino acids that increase the interaction between the proteins.

Two simple mutations to Model C were introduced to alter the interaction energy between V13 and VEGF_165_: a Pro98Tyr substitution, which increased the interaction energy, and an Arg97Alasubstitution, which reduced the interaction energy (Table 2). The control complex was used as a reference. Figure 2 shows a map of the contact points for Model C of the V13-VEGF_165_ complex. Pro98 was modified to increase the interaction energy, which was initially low. Upon substituting Tyr for Pro98, a more favorable hydrogen bond formed between the new Tyr98 of V13 and Glu51 of VEGF_165_. In addition, the interaction between Arg90 of V13 and Leu84 of VEGF_165_ was improved. The loss of strength in the contact between Lys92 of V13 and VEGF_165_ was compensated by the appearance of a new contact between Asn93 of V13 and VEGF_165_.

**Table 2:** Interaction energy of mutants chosen for the V13-VEGF_165_ complex

Models	E_total_	E_vdw_	E_elect_	E_HB_	SASA	N° atoms	E_total_/N° atoms	E_total_/SASA
VEGF_165_-V13	-124.10	-83.96	-61.22	-15.35	727.4	4279	-0.029	0.1706
Mutant 1 P98Y	-131.47	-79.07	-70.83	-18.96	708.6	4286	-0.031	-0.1855
Mutant 4 R97A	-93.56	-70.24	-40.99	-12.32	627.2	4269	-0.021	-0.1491

The energy and occluded surface values were calculated for the minimal average structure in the last 500 ps of dynamics.

E_total_: total energy; E_vdw_: Van der Waals energy; E_HB_: energy hydrogen bonds; SASA: solvent-accessible surface area.

**Figure 3:**
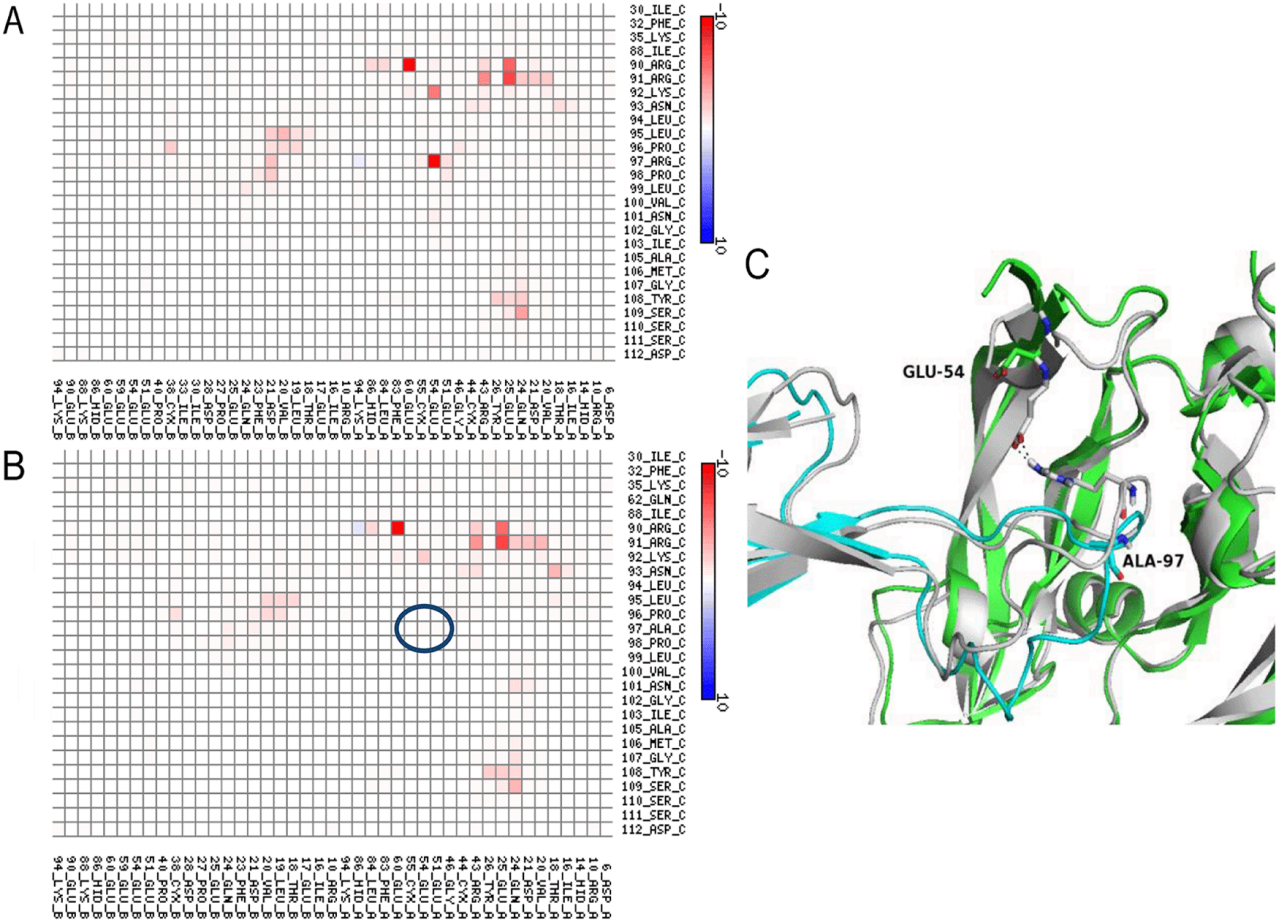
Interaction map for Mutant 2 (Arg97Ala) within the VNAR V13-VEGF_165_ complex Interactions between V13 (vertical axis) and VEGF_165_ (horizontal axis) are shown for the parental **(A)** and mutated **(B)** forms. The color scale is a function of the value of the interaction energy: redder shades indicate more favorable interactions, while bluer tints are less favorable. **(C)** Representation of the interaction at position 97 of V13 and position 60 of VEGF_165_ before (white cartoon) and after ( V13 green cartoon and VEGF_165_ cyan cartoon) mutation. The blue circles show the amino acids that decrease the interaction between the proteins.

Figure 3 shows the contact map for the V13-VEGF_165_ complex after substituting Ala for Arg97. The interaction between Arg97 of V13 with Glu54 of VEGF_165_, which was only seen in Models A and C, is one of the strongest bonds between the two proteins. In both models V13 binds VEGF_165_ similarly; thus, their mutation to Ala decreased the interaction energy (one hydrogen bond was removed). This highlights the importance of Arg97 for the V13-VEGF_165_ interaction.

### VNAR expression

**Figure 4:**
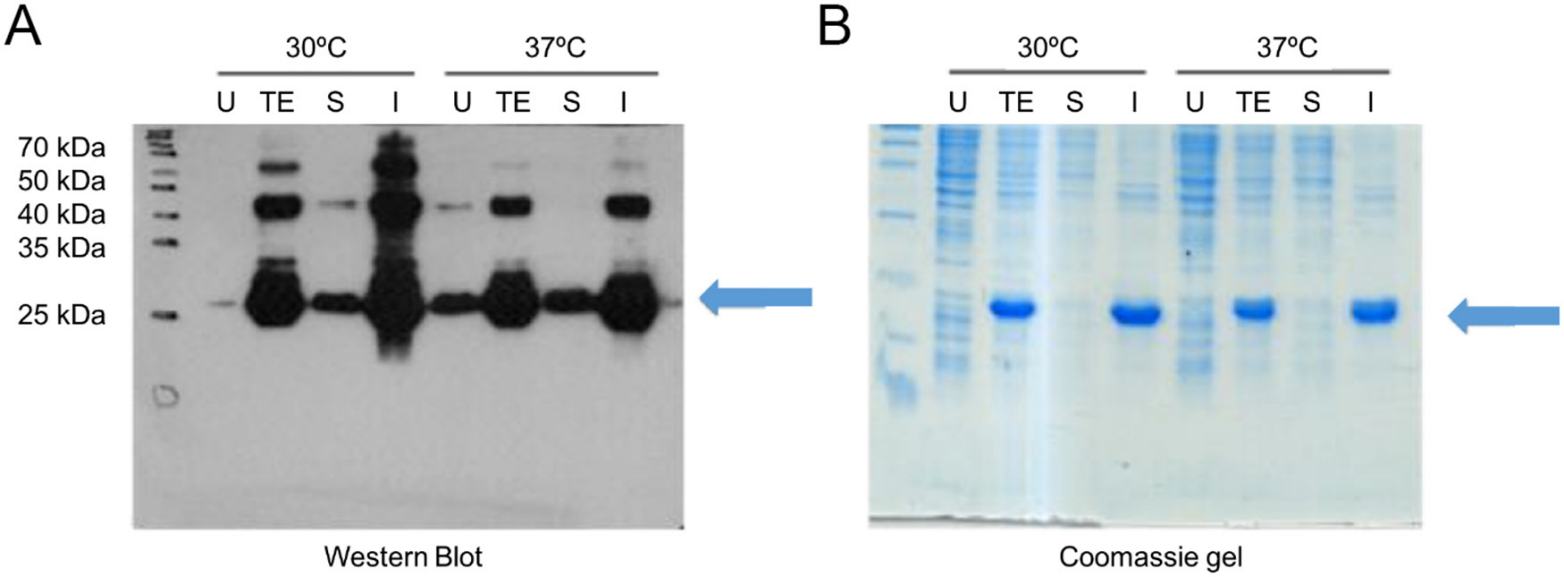
Expression of anti-VEGF_165_ VNAR In each case, lanes correspond to total extract without induction (U), total extract after the induction time (TE), soluble fraction after cell lysis (S), and insoluble fraction after lysis (I). Equivalent amounts of cell extract were loaded onto the gels. **(A)** Western blot and chemiluminescence. **(B)** Coomassie-stained gel.

**Figure 5:**
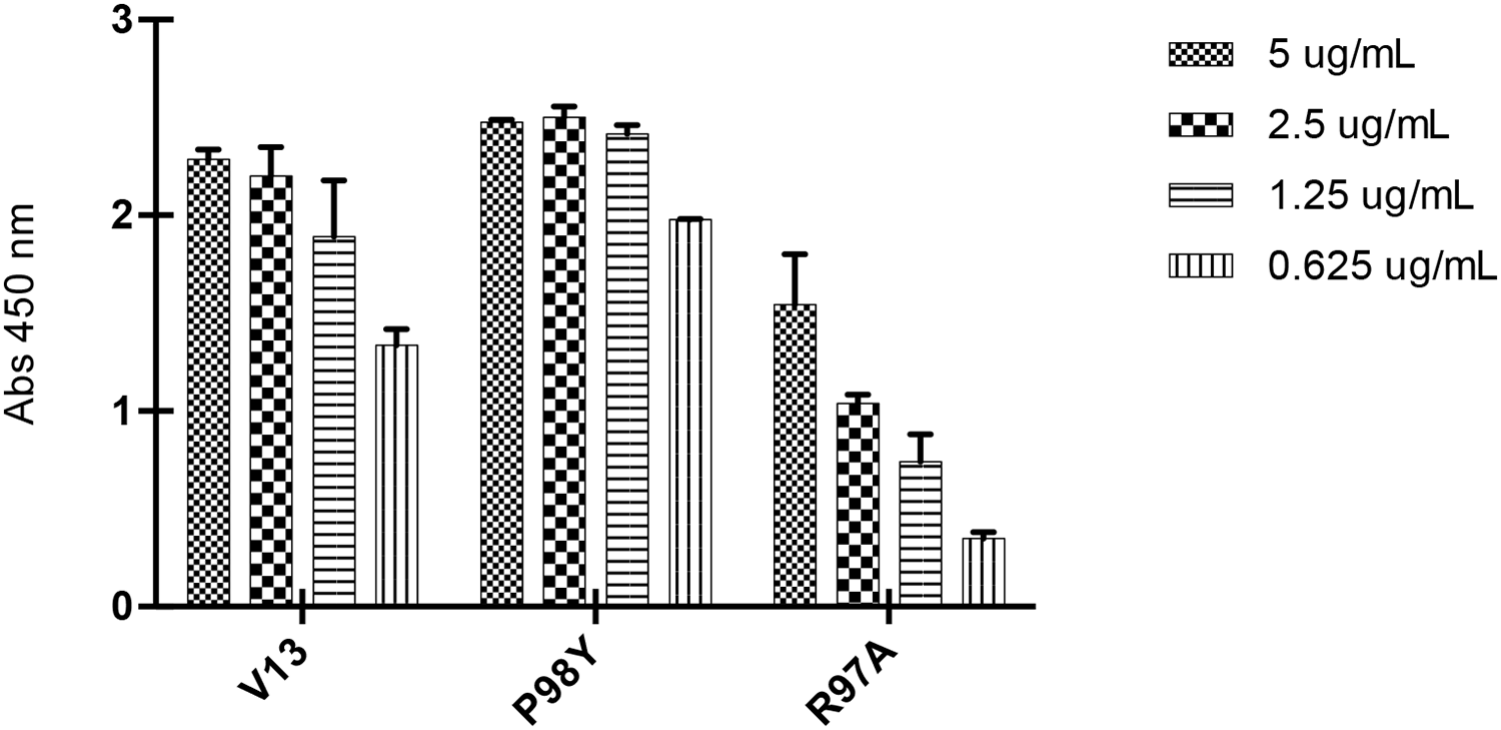
ELISA titration of *in silico*-generated VNARs Decreasing concentrations of each VNAR were tested (5, 2.5, 1.25 and 0.625 μg/mL). Pro98Tyr shows better recognition than V13 at each concentration; while Arg97Ala shows weaker recognition that V13 at each concentration.

The genes for the Pro98Tyr and Arg97Ala VNAR mutants and parental V13 were expressed in *E. coli* BL21 (DE3). Figure 4 shows the analysis of the expressed proteins, which were purified using IMAC, with final yields of 7.35 mg/L (V13), 3.48 mg/L (Pro98Tyr) and 13.86 mg/L (Arg97Ala). Using ELISA plates coated with VEGF_165_, we found that after the wells were blocked with 3% BSA, clone Pro98Tyr recognized VEGF_165_ better than parental V13 at all concentrations (Figure 5). This is consistent with model described above.

### *In vitro* angiogenesis assay

**Figure 6:**
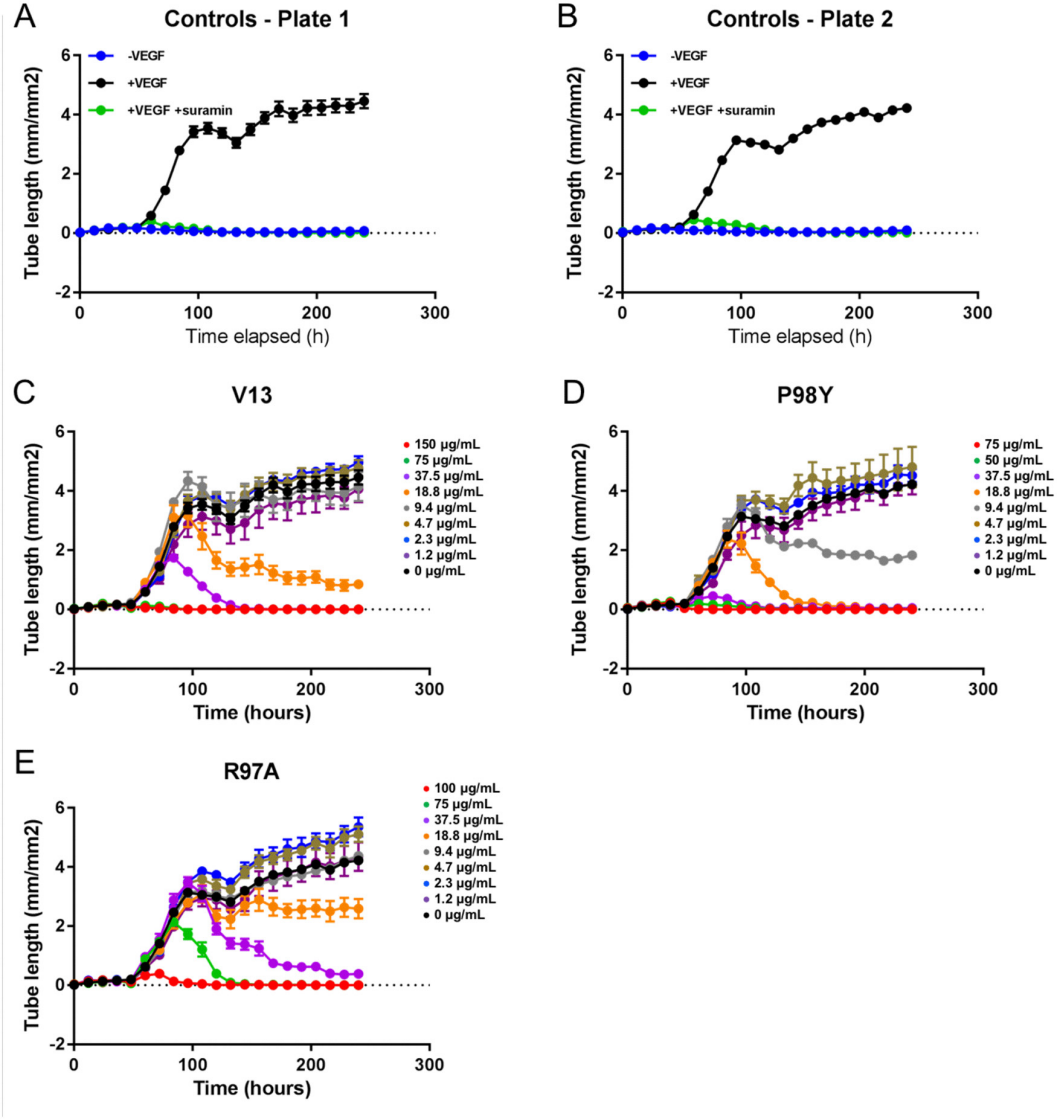
Effects of *in silico* designed mutants and V13 VNARs on HUVEC-vessel tube length **(A** and **B)** Forty-eight hours after seeding, cells were left untreated (-VEGF_165_), treated with 4 ng/mL VEGF_165_ (+VEGF_165_), or treated with 4 ng/mL VEGF_165_ and 100 μM suramin (+VEGF_165_+suramin). **(C-E)** Forty-eight hours after seeding, cells were treated with 4 ng/mL VEGF_165_ and the indicated concentration of each test VNAR. Tube length was recorded for 240 hours (A-E).

**Figure 7:**
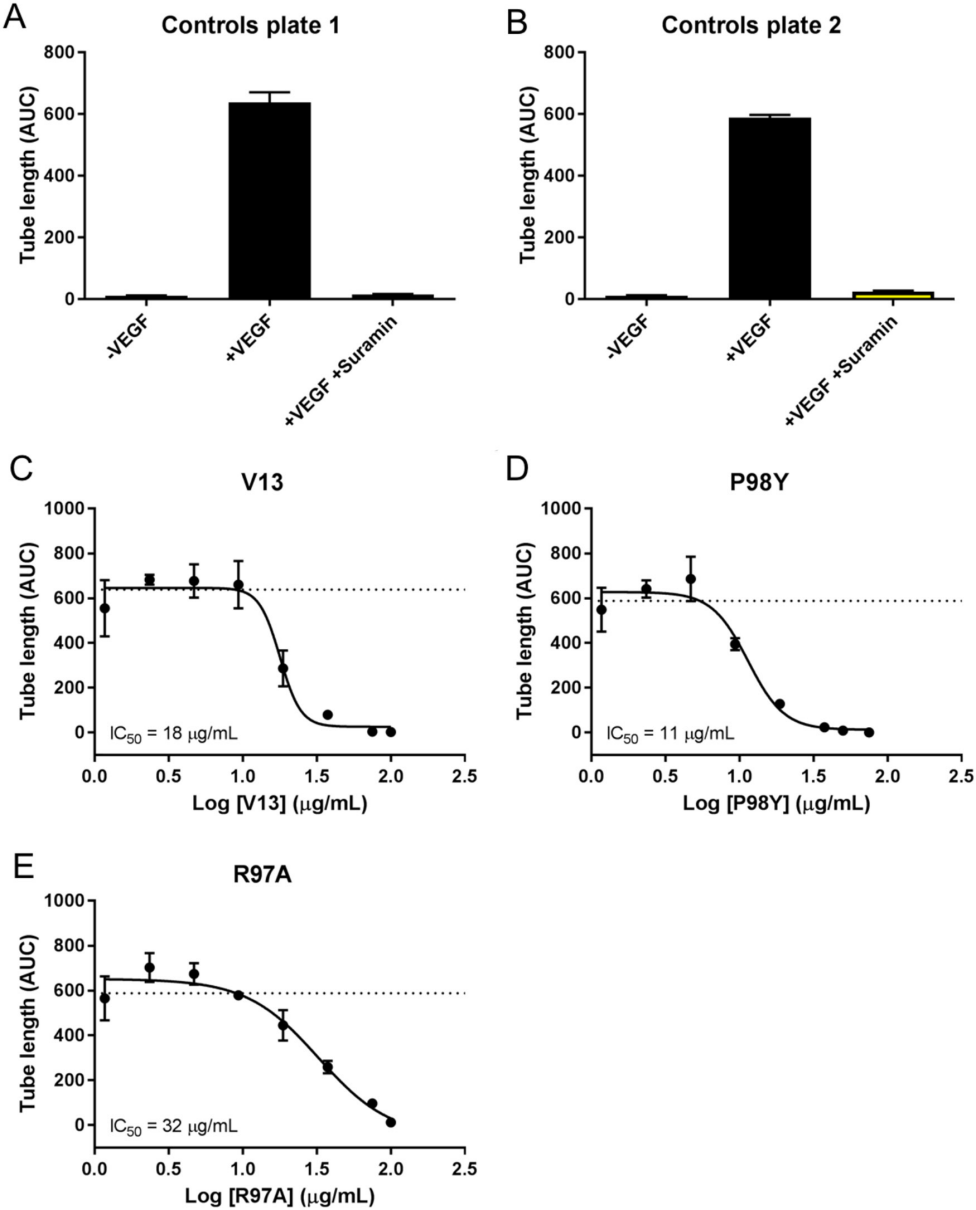
AUC analysis of test VNARs effect on HUVEC vessel tube length AUCs for each time point were calculated for vessel tube length using GraphPad Prism. Curve fitting **(B-E)** was performed using nonlinear regression (4 parameters), and the average of the +VEGF_165_ controls is indicated by a dashed line.

Vascular tube formation assays were run using a co-culture system with GFP-expressing HUVECs and NHDFs. Forty-eight hours after seeding, the cells were treated with VEGF_165_ at 4 ng/mL and with the test compounds at eight different concentrations (Table 3). The formation of vessel tubes by fluorescently-labeled HUVECs was measured using live-cell imaging, and the effects of the VNARs on this process were assessed. The inhibition of vascular tube formation by the VNARs, based on vascular tube length, is shown in Figure 6, while areas under the curve (AUCs) are shown in Figure 7.

**Table 3:** Summary of final assay concentrations (FACs) of individual VNARs

VNAR	Compound and buffer titrations								
V13	100	75.0	37.5	18.8	9.4	4.7	2.3	1.2	Compound FAC (μg/mL)
P98Y	75	50.0	37.5	18.8	9.4	4.7	2.3	1.2	Compound FAC (μg/mL)
R97A	100	75.0	37.5	18.8	9.4	4.7	2.3	1.2	Compound FAC (μg/mL)

**Figure 8:**
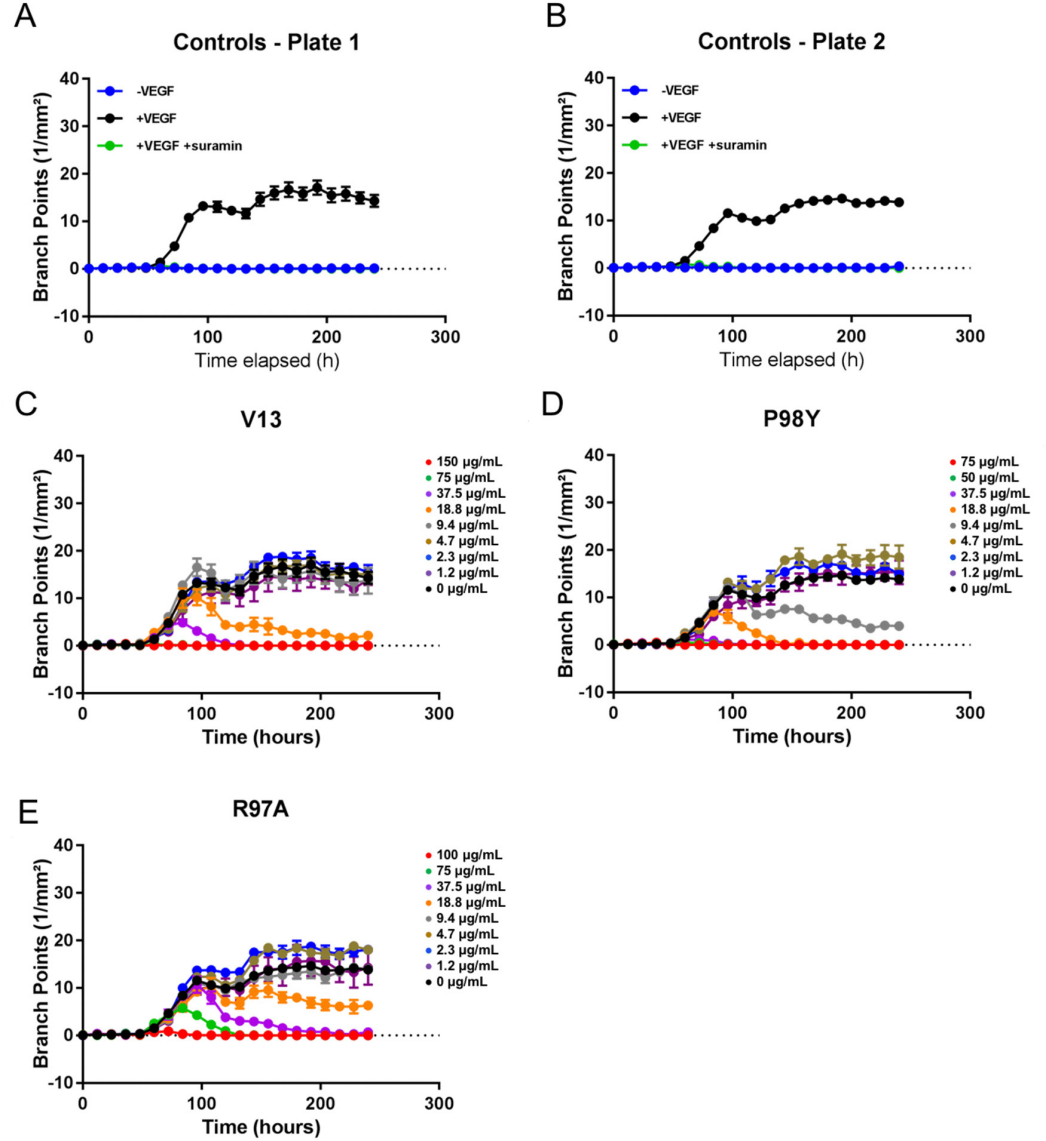
Effect of test VNARs on HUVEC-vessel network branching **(A** and **B)** Forty-eight hours after seeding, HUVECs were left untreated (-VEGF_165_), treated with 4 ng/mL VEGF_165_ (+VEGF_165_), or treated with 4 ng/mL VEGF_165_ and 100 μM suramin (+VEGF_165_+suramin). Network branching was recorded for 240 hours. **(C-E)** Forty-eight hours after seeding, cells were treated with 4 ng/mL VEGF_165_ and the indicated concentration of each test VNAR. Network branching was recorded for 240 hours.

**Figure 9:**
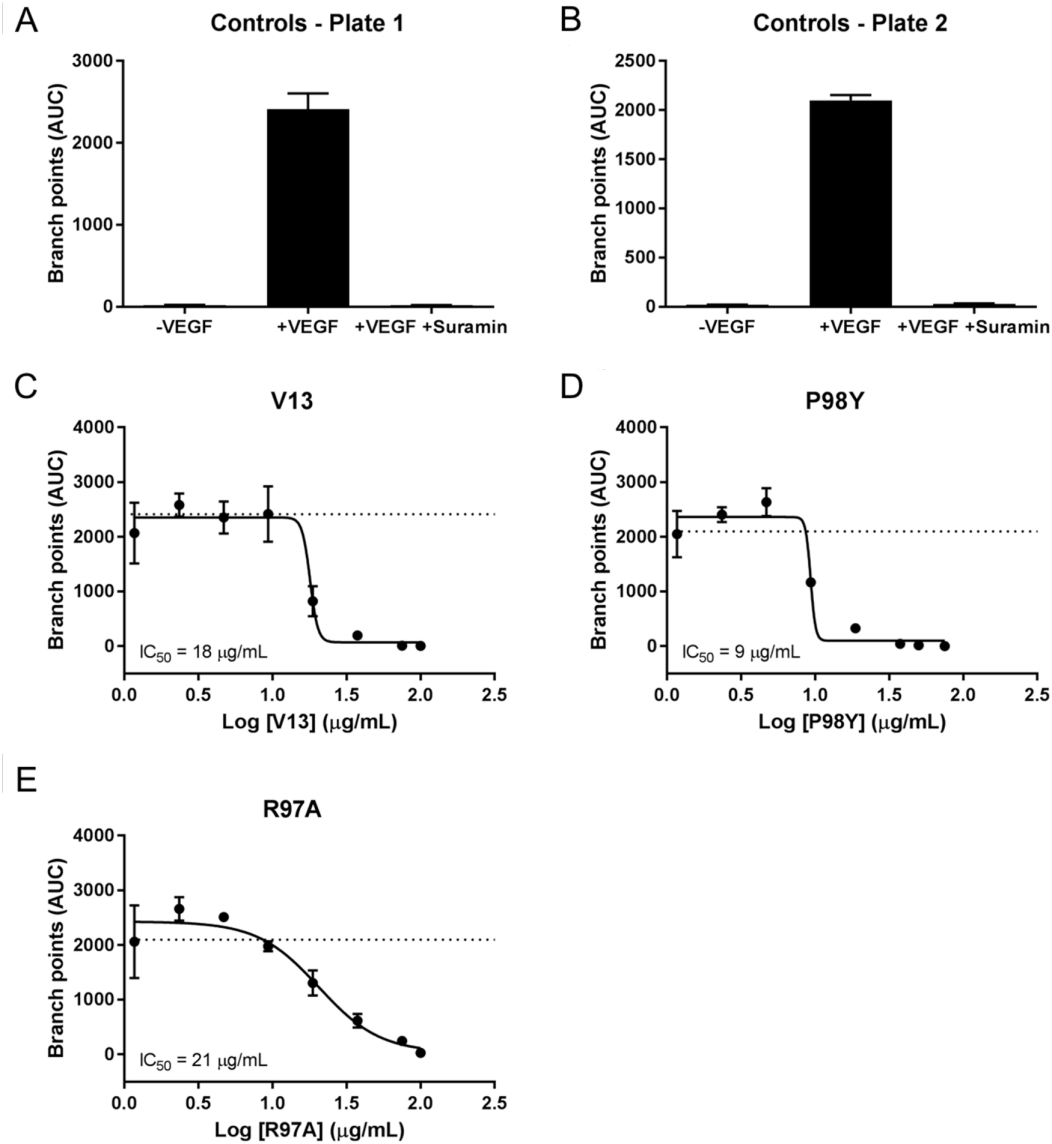
AUC analysis of test effect of VNARs on HUVEC-vessel branching AUCs for each time point were calculated for branch points using GraphPad Prism. Curve fitting **(A-E)** was carried out using nonlinear regression (four parameters), and the average of the +VEGF_165_ controls is indicated by a dashed line.

VEGF_165_ stimulation also induced HUVEC vessel tube branching, which was inhibited by suramin, a VEGF_165_ receptor 2 inhibitor (positive control). Parental VNAR V13 as well as the two mutants all dose-dependently decreased tube length and network branching. In these analyses, the V13 Pro98Tyr mutant was especially effective for angiogenesis inhibition (Figure 8). The AUCs are shown in Figure 9. To confirm these results, additional assays were performed in which branching and tube formation by endothelial-cell spheroids, stimulated with VEGF was measured. As shown in Supplementary Figure 3, despite using a different model, the same pattern of results was obtained: Pro98Tyr inhibited branching and lengthening of endothelial cell tubes more effectively than the parental V13.

### *In vivo* inhibition of vessel and tumor growth

**Figure 10:**
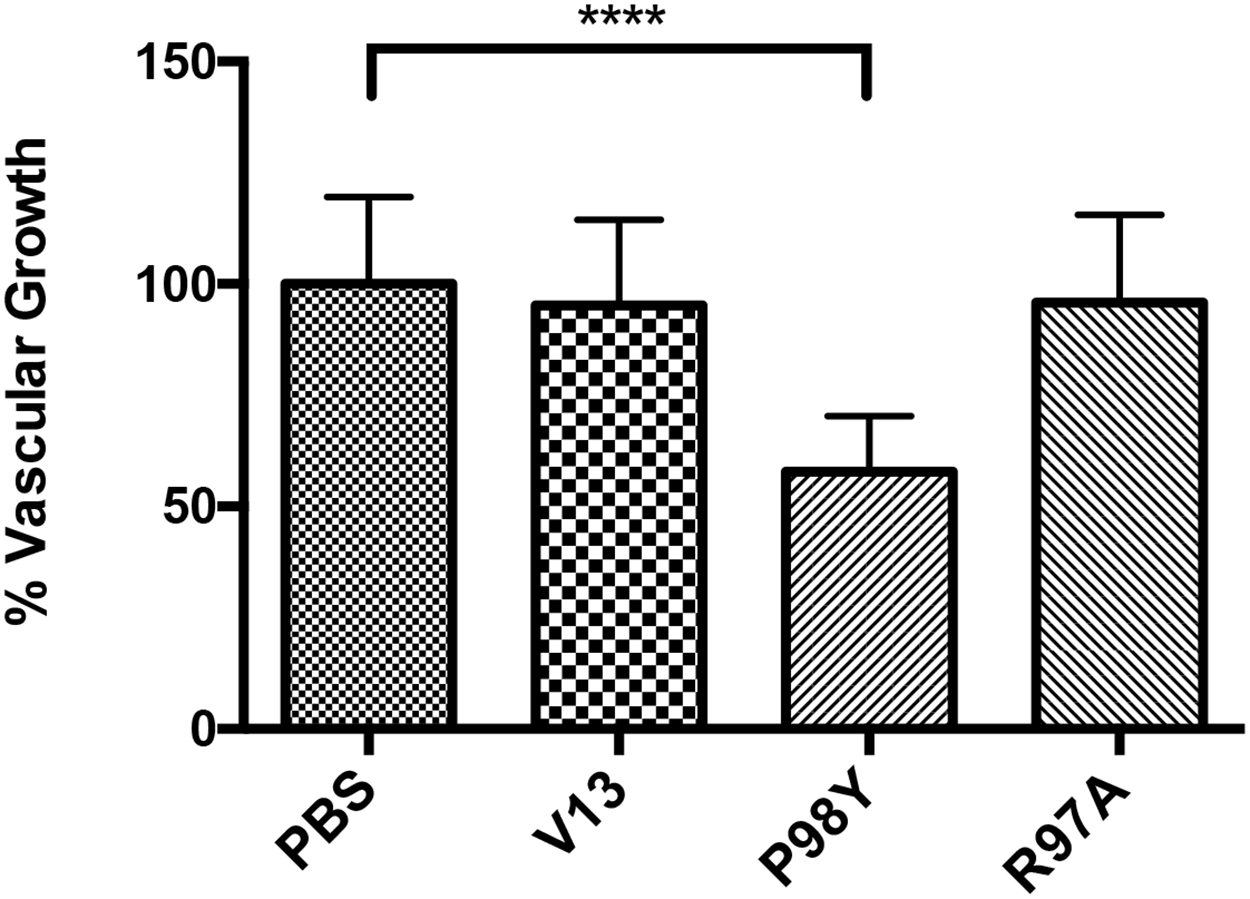
Anti-VEGF_165_ activity shown inhibition tumor-related vascular growth by VNARs After 18 days of treatment, effects on capillary growth around the tumor were measured. Only Pro98Tyr elicited a significant difference from untreated controls (P <0.0001, one ANOVA-way). No inhibitory effect was detected with Arg97Ala or with parental V13.

VNARs were tested for their ability to inhibit vascular growth *in vivo* in a mouse-tumor model with lung-cancer cells. Treatment with six doses of the Pro98Tyr mutant over 18 days significantly reduced capillary growth around the tumor as compared to untreated controls (P <0.0001, one ANOVA-way) (Figure 10). With Arg97Ala, the inhibition was comparable to that obtained with parental V13.

V13, Pro98Tyr and Arg97Ala were also tested for their ability to inhibit tumor growth *in vivo* in the same mouse-tumor model. After day 18, each tumor was collected and weighed. Table 4 shows the normalized average weight (n=8) from each treated group, where PBS (untreated mice) represents the maximum recorded weight (100%). Only mice treated with Pro98Tyr showed a significant reduction of tumor weight (p=0.02). V13 also tended to reduce tumor weight, but the effect was not significant (p=0.06). These results are consistent with all the previous results and provide a solid basis for continuing to use this VNAR for future development.

**Table 4:** Comparison of normalized tumor weight among treatments

Clone	Normalized tumor weight	Standard deviation	p value
PBS	1.0	0.23	
V13	0.63	0.31	0.06
R97A	0.76	0.24	0.16
P98Y	0.71	0.36	0.02

## DISCUSSION

*In silico* analysis generated one positive VNAR with a single mutation (Pro98Tyr) and one negative VNAR (Arg97Ala). The substitution of Ala for Arg at position 97 decreased the recognition of the VNAR by VEGF_165_, as expected. Conversely, substituting Tyr for Pro at position 98 improved these properties. For the angiogenic effect on HUVEC-vessel tube length, the IC_50_ of V13 decreased from 18 to 9 μg/mL as a result of this amino acid change (Table 5). Pro is an aromatic nonpolar amino acid that does not form hydrogen bonds, whereas Tyr is an ionic nonpolar amino acid that can generate such bonds and increase protein-protein interactions. Although Tyr is an aromatic amino acid, as is proline, the –OH group on the aromatic ring of Tyr stabilizes protein-protein interactions.

**Table 5:** Comparison of IC_50_ values from angiogenesis assays

Clone	IC_50_ angiogenesis: tube length (μg/mL)	IC_50_ angiogenesis: network branching (μg/mL)
V13	18	18
P98Y	9	11
R97A	21	33

Concentrations that were required to inhibit network tube length proliferation and network branching.

The experimental evaluation of the *in silico* designed VNARs, resulted in a predicted response. Pro98Tyr recognized VEGF_165_ more effectively than parental V13, with correspondingly better inhibition of HUVEC vessel tube length (IC_50_ = 18 μg/mL for V13 vs 11 μg/mL for Pro98Arg) and branch point formation in HUVEC vessels (IC_50_ = 18 μg/mL for V13 vs 9 μg/mL for Pro98Arg). By contrast, Arg97Ala substitution reduced VNAR activity as compared to V13 in all the *in vitro* tests. *In vivo*, moreover, Pro98Arg showed greater inhibition of tumor vascularization than parental V13. These results validate our *in silico* designed mutation system.

Clearance of small antibody fragments is reportedly faster than clearance of larger fragments or whole antibodies [19–24]. With a molecular weight of 13 kDa, VNARs are small. The plasma half-life is 110 h for whole antibodies, 5 h for a minibody and 2 h for a scFv, but only 3 min for a V domain (such as V13) [20]. Consequently, a single-domain VNAR cannot be used for chronic treatment. Nonetheless, Pro98Tyr exerted significant inhibitory effects on vascular tube length and branch point formation and on tumor size *in vivo*. Future assays will enable determination of the real plasma half-life of Pro98Arg, while preclinical trials will be needed to determine whether a single-domain VNAR can be used as a therapeutic drug or if modification to increase plasma half-life is necessary.

In summary, we generated a more potent VNAR (Pro98Tyr vs parental V13) using a computational model to introduce *in silico* designed mutations. The new VNAR could potentially be used as a novel drug to neutralize VEGF_165_. Given its potency, it is anticipated that less protein, eliciting fewer side effects, will be required.

## MATERIALS AND METHODS

### *In silico* strategy

#### VNAR V13 protein modeling

The structure of VNAR V13 was modeled by threading using I-TASSER (Iterative Threading Assembly Refinement) [25–27]. Fragment VNAR V13 was described previously [18].

#### Molecular dynamics (MD)

An MD simulation was performed using the AMBER suite [28]. Standard atomic charges and radii were assigned according to the AMBER ff03r1 force field, and the system was immersed in a cubic box of TIP3P water molecules [29] that was sufficiently large to ensure that the shortest distance between the receptor and the edge of the box was greater than 12 Å. Counterions were also added to maintain electroneutrality. Three consecutive minimizations were performed involving: (i) only hydrogen atoms; (ii) only water molecules and ions; and (iii) the entire system. The resulting structure was simulated in the NPT ensemble (N, total number of atoms; P, pressure; T, temperature) using periodic boundary conditions and the particle mesh Ewald method to deal with long-range electrostatic interactions. The system was then heated and equilibrated in 2 steps: (i) 20-ps MD, heating the entire system from 100 to 300 K; and (ii) equilibration of the entire system for 100 ps at 300 K. This equilibrated structure was the starting point for a 100-ns MD simulation at a constant temperature (300 K) and pressure (1 atm). The SHAKE constraint algorithm was used to keep the bonds that involved H atoms at their equilibrium length, allowing a 2 fs step for the integration of Newton's equations for motion.

To identify the most stable structure over a 100-ns simulation, cluster analysis was performed with the ptraj module in the AMBER 12 package. Finally, we calculated the time-averaged structure that corresponded to the most populated cluster, which included snapshots along 40 ns, incorporating mass-weighted positional fluctuations and root-mean-square deviations with the ptraj module. This structure was minimized in a vacuum under the AMBER ff03r1 force field, without periodic boundary conditions, over 1000 steps to alleviate any clashes that might have originated from averaging the coordinates. This refined, time-averaged structure was the ligand (VNAR V13) used for docking to the crystal structure of VEGF_165_ (Protein Data Bank entry 1VPF) [30], which was used as a receptor and was previously refined by MD over 6 ns.

#### Protein-protein interaction

To examine the binding site of the V13 for VEGF_165_, a protein-protein docking protocol was applied using the ClusPro web tool [30, 31], which is based on surface complementarities. The resulting structures of the complexes were filtered to select those with good electrostatic and desolvation free energies for further clustering. The output was a short list of putative complexes, ranked according to their clustering properties [31]. The PyMOL program [32] was used to visualize the selected models. The four complexes with the highest binding affinities, and differing in binding orientation, were chosen as potential VEGF_165_-V13 complexes. These candidates were examined further in MD simulations using the AMBER suite. The predicted 3D structures were solvated in a water box with a minimum distance of 12 Å between the complex boundaries and the edges of the box. Na^+^ and Cl^-^ ions were added to neutralize the system. The equilibrated systems were then subjected to a 20-ns MD simulation in an isothermal-isobaric (NPT) ensemble per the method described above. The four MD trajectories were further analyzed using MM-ISMSA [33] to estimate the total free energy of the binding and the relative contributions of the binding-site residues.

#### V13 mutation and MD simulation analysis

The critical amino acid residues in VEGF_165_ and V13 that contributed to their interaction in the selected complexes were identified as described and mutated. V13 mutations were modeled, and MD simulations were performed to study the impact of these mutations on the interaction between complexes. Four new VEGF_165_-V13 complexes that harbored these mutations were examined. PyMOL was used to mutate the models [32].

The stability and free energies were analyzed through MD simulation using the AMBER 12 package. The mutated structures were solvated in water molecules inside of a box with a minimum distance of 12 Å between the complex boundaries and the edges of the box. Na^+^ and Cl^-^ ions were added to neutralize the system, and the equilibrated systems were then subjected to a 5-ns MD simulation in the isothermal-isobaric (NPT) ensemble per the method described above. The four MD trajectories were further analyzed using MM-ISMSA to estimate the total free energy of the binding and the relative contributions of the binding-site residues.

### *In vitro* strategy

#### VNAR expression

All genes were synthetized by IDT Inc. After optimizing the sequences from V13 and each mutant for expression in *E. coli*, the genes were subcloned into pET-28a(+) vector (Novagen®) using the NcoI and BamHI restriction sites. The plasmids were electroporated into BL21(DE3) cells (Invitrogen Life Technologies) and stored at -80°C.

All proteins were expressed in 50-mL cultures at 30°C for 18 h and at 37°C for 4 h. Expression was induced using 0.8 mM IPTG. After induction, the cells were harvested, and protein expression was analyzed using SDS-PAGE, Coomassie staining, and western blotting with anti-6x histidine.

All proteins were purified using immobilized metal affinity chromatography (IMAC). Two types of extracts were used: the soluble fraction (cytoplasmic) and the insoluble fraction (inclusion bodies). Inclusion bodies were treated with denaturants and refolded on-column. The two fractions were then pooled to obtain as much protein as possible and evaluated by SDS-PAGE.

#### ELISA assay

A total of 250 ng of VEGF_165_ were added to each well of an ELISA plate. The plate was incubated for 1 h at 37°C, and the remaining VEGF_165_ was removed by washing. Then, 150 μL blocking solution (3% BSA on PBS-1X) were added to each well and incubated for 1 h at 37°C. The blocking solution was removed, and the wells were washed 3 times with PBS-Tween (PBST). Next, 50 μL of each VNAR were added to each well in triplicate at various concentrations (5, 2.5, 1.25 and 0.625 μg/mL) and incubated for 1 h at 37°C. BSA (1%) was used as a negative control. The solution was decanted, the plate was washed three times with PBST, and 50 μL of anti-HA-HRP (diluted 1:1000 in 1% BSA-1X PBS) were added to each well. The plate was incubated for 1 h at 37°C. The solution was removed, and the wells were washed three times with PBST. TMB substrate (50 μL) was added, and the plate was incubated at room temperature for 15-30 min or until the desired color developed. The reaction was stopped with 50 μL of 2 M sulfuric acid, and the absorbance was read at 450 nm.

#### Angiogenesis assays

Normal human dermal fibroblasts (NHDFs) and green fluorescent protein (GFP)-labeled human umbilical-vein endothelial cells (HUVECs) were purchased from Essen Bioscience as part of the CellPlayer 96-Well Kinetic Angiogenesis PrimeKit (Essen Bioscience #4452). Seeding medium, growth medium, assay medium, and their respective supplements were obtained as part of the same kit. VEGF_165_ and suramin were purchased as part of the CellPlayer Angiogenesis PrimeKit VEGF_165_/suramin supplement (Essen Bioscience #4437). Plates (96-well) were obtained from Corning (#3595). Resazurin and Dulbecco's PBS were purchased from Sigma, UK.

For cell seeding, on day 0, one cryogenic vial of NHDF cells was thawed and suspended into 12 mL of complete seeding medium. Aliquots (100 μL) of the suspension were then added to the wells of a 96-well plate, and the cells were incubated at room temperature for 1 h. A cryogenic vial of HUVECs was thawed and suspended in 12 mL complete seeding medium, after which the HUVECs (100 μL per well) were seeded into the same plate as the NHDFs, and the cells were incubated at room temperature for an additional 1 h. The plate was then imaged on an IncuCyte ZOOM and scanned using the “Tiled FOV” scan type. After 24 h, the culture medium in each well was replaced with 150 μL growth medium.

VEGF_165_ was added to the assay medium to a final concentration of 4 ng/mL. Serial dilutions of VNARs were prepared using VEGF_165_-containing assay medium at the concentrations shown in Table 3. Then, 150 μL of the medium that contained the VNARs were added to the cells. To untreated wells, medium without VEGF was added. To the no-compound wells, medium with VEGF_165_ was added. As a positive control, 100 μM suramin was added to medium containing VEGF_165_. Assay media, VEGF_165_, and test VNARs were added to the cells on days 2, 5, 7, 9, and 11. Eight wells (no VNAR) or three wells were seeded to assess tube length and branch points. VNAR concentrations were used as follows: V13 (150, 75, 37.5, 18.8, 9.4, 4.7, 2.3, 1.2 and 0 μg/mL), P98Y (75, 50, 37.5, 18.8, 9.4, 4.7, 2.3, 1.2, and 0 μg/mL) and R97A (100, 75, 37.5, 18.8, 9.4, 4.7, 2.3, 1.2 and 0 μg/mL).

Cells were imaged on an IncuCyte ZOOM with data points recorded every 12 h for analysis. IncuCyte ZOOM software was used to calculate tube length and branch points. Filters were used to specify a minimum tube length of 0.2 mm and a minimum tube width uniformity of 0.65 mm. Area-under-the curve values for network length and branch points were calculated using GraphPad Prism.

### Angiogenesis assay on a 3-dimensional HUVEC spheroid cell model

HUVEC cells (Cell Applications, Inc) were cultivated from the second to fourth passage, at most, in endothelial cell growth medium (ECGM). The cells were cultured until 80% confluence was reached. The cultured cells were trypsinized and resuspended in ECGM medium supplemented with 20% metocel, in a 400 cells/100 μl dilution, to generate spheroids of approximately 400 cells. The cell suspension was distributed in a 96 well U bottom non-adhesive plates and were incubated at 37°C with 5% CO2 for 24h. This incubation allowed the formation of the spheroid [34]. The spheroids were recovered from the plates, centrifuged at 300 x g for 10 min and were carefully resuspended in a collagen solution (2 mg/mL) at 4°C, pH 7.4 (adjusted with 0.2 N NaOH), mixed with 1:1 metocel + 20% SFB + 10 mM of HEPES. The solution with the spheroids was quickly distributed in a 24-well flat-bottom non-adhesive plate, 1 mL of solution was deposited in each well. Each mL of the solution contained approximately 40 spheroids. The plate was placed at room temperature for 10 min and was then incubated at 37°C for 30 min to allow the gelling of the collagen. The different treatments (100 μl) were then added: 1) basal medium (MB, control group); 2) VEGF (50 ng) + MB; 3) VEGF (50 ng)+V13 antibody (10 μg); 4) VEGF (50 ng)+P98Y antibody (10 μg). The plate was incubated in a humidified incubator for 4 h at 37°C with 5% CO2. Furthermore, 10 μg of anti-VEGF antibody (in 50 μL of PBS) was added to the corresponding treatments and the plate was incubated under the previously mentioned conditions for a total of 24 h. The spheroids were fixed by adding 1 mL of 10% formalin. The *in vitro* angiogenesis was quantified digitally by measuring the length of the sprouts instead of ramifications that grew on the spheroid. The measurement was recorded using an inverted microscope (Olympus, Germany) and a digital image software (Image Pro®). For each group 20 spheroids were measured. Differences between treatment groups were tested by unpaired Student's t test. P values <0.05 were considered statistically significant.

### *In vivo* strategy

Nestin-driven GFP (ND-GFP) mice (6-10 weeks old), which express GFP in nascent blood vessels [35], were used for the *in vivo* assays at AntiCancer, Inc. (San Diego, California, USA). Under Assurance #A3873-1from the National Institutes of Health, anesthesia and analgesics were used for all surgical experiments. Animals were anesthetized by subcutaneous injection of 0.02 mL of 20 mg/kg ketamine, 15.2 mg/kg xylazine, and 0.48 mg/kg acepromazine maleate. Responses of the animals were monitored during surgery to ensure adequate depth of anesthesia. The animals were observed on a daily basis and humanely sacrificed through CO_2_ inhalation when they met the following humane endpoint criteria: severe tumor burden (more than 20 mm in diameter), prostration, significant body weight loss, difficulty breathing, rotational motion, and body temperature drop. Animals were housed in a barrier facility on a high-efficiency particulate arrestance-filtered rack under standard conditions of a 12-h light/dark cycle. The animals were fed an autoclaved laboratory rodent diet. Briefly, eight ND-GFP mice were used per group. Murine Lewis lung cancer cell lines stably expressing red fluorescent protein (RFP) were used for cancer cell implantation in the foot pad (5×10^5^ cells in 25 μL). Treatment started 3 days after cancer-cell implantation. All VNARs were administrated intraperitoneally twice per week for a total of 6 doses over 18 days. Each dose contained 27 μg of VNAR in 180 μl of PBS. After day 18, the implanted foot pad of the mice in each group was imaged for GFP-expressing blood vessels. The vessel density was calculated as the total length of the blood vessels divided by the observed area. Each tumor was collected and weighed (g). This project was also approved by the CICESE Bio-Ethical Committee, approval number 2014/03.

### Statistical analysis

All data were analyzed using one ANOVA-way or unpaired Student's t test. Numbers of repetitions and animals are given in the text and/or figures.

## SUPPLEMENTARY MATERIALS FIGURES AND TABLES


