# Supplementary material for: In silico-designed mutations increase variable new-antigen receptor single-domain antibodies for VEGF165 neutralization
Source: Oncotarget. 2018 Jun 15;9(46):28016–29. doi: 10.18632/oncotarget.25549 (PMC6021326; doi:10.18632/oncotarget.25549)
Supplement: Supplementary file 1 [file oncotarget-09-28016-s001.pdf]

In silico-designed mutations increase variable new-antigen receptor single-domain antibodies for $VEGF_{165}$ neutralization

SUPPLEMENTARY MATERIALS

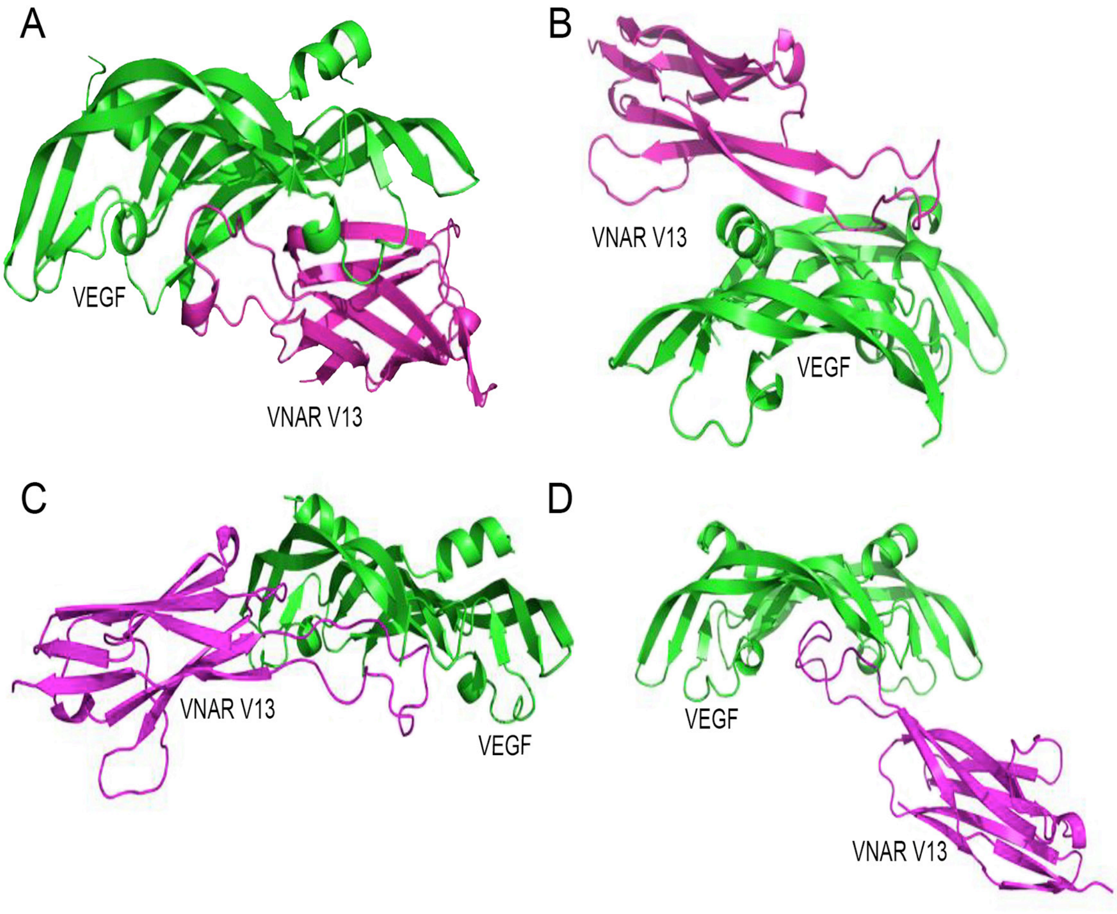

Supplementary Figure 1: Models of VNAR V13-$VEGF_{165}$ protein-protein interactions. V13 (magenta)-$VEGF_{165}$ (green): **(A)** Model A, **(B)** Model B, **(C)** Model C, **(D)** Model D.

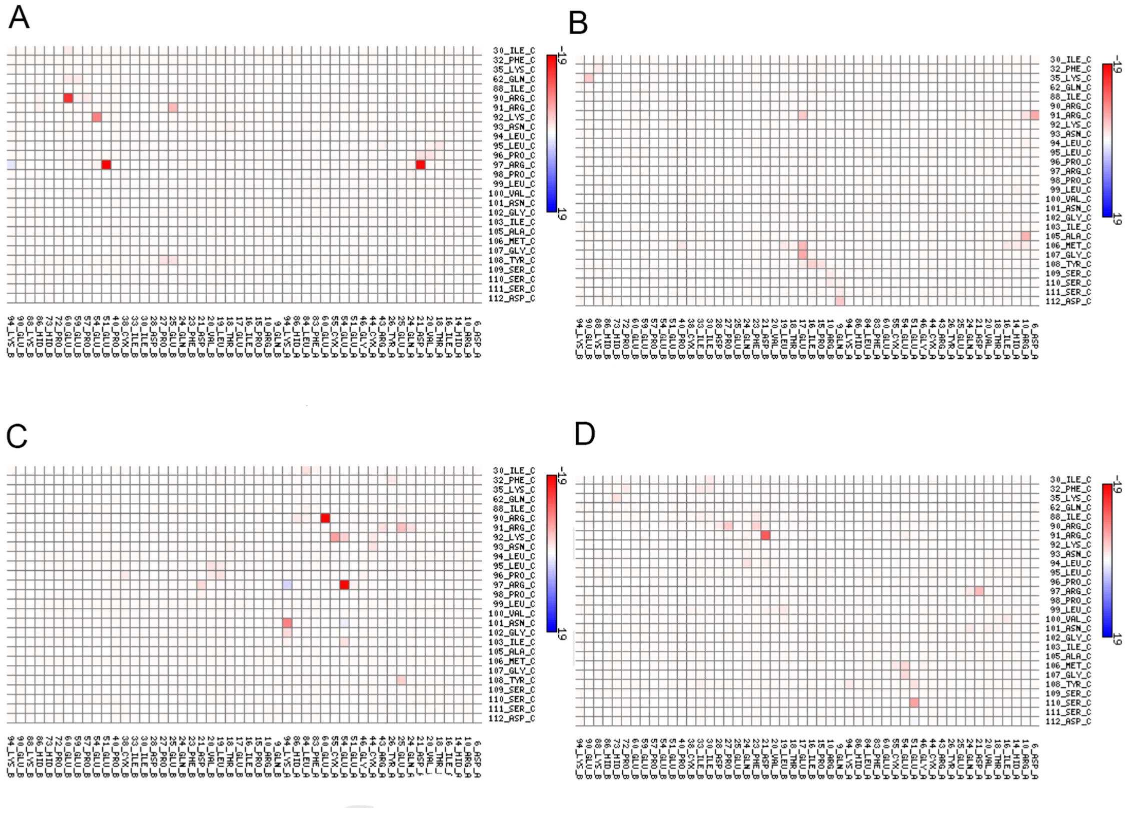

Supplementary Figure 2: Interaction maps of VEGF$_{165}$-V13 complexes. Shown areinteractions between V13 (represented in vertically) and VEGF$_{165}$ (represented horizontally) complexes. The color scale is based on the value of the interaction energy: redder indicates more favorable interactions, while the bluer tint is less favorable. **(A)** Model A, **(B)** Model B, **(C)** Model C, **(D)** Model D.

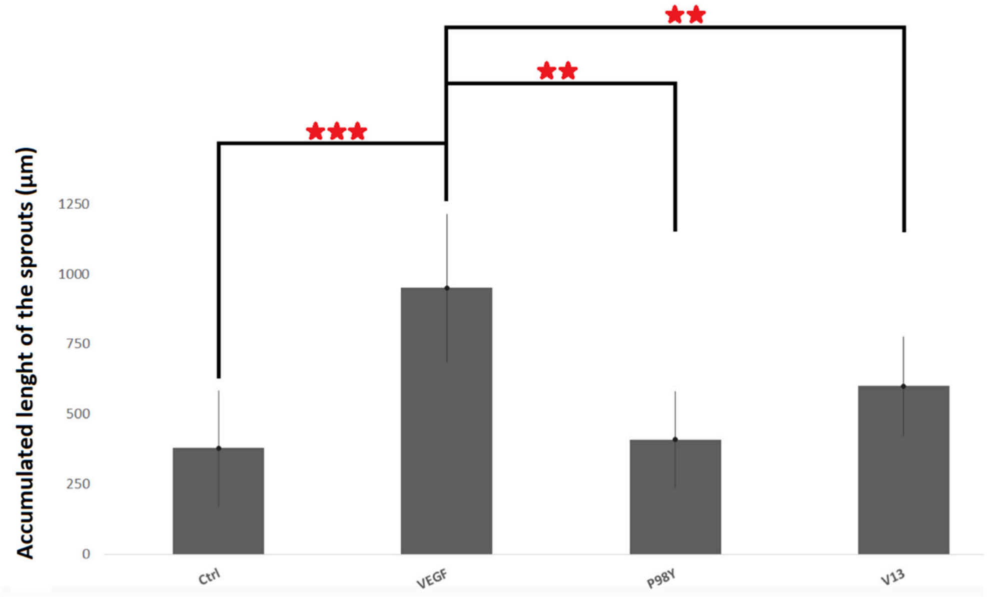

Supplementary Figure 3: Measurement of branch length in endothelial cell spheroids stimulated with $VEGF_{165}$. Statistical differences were observed with VEFG when compared to the control group, P98Y and V13. A highly significant statistical difference ($P<0.001^{***}$) was observed between the control group and the VEFG group. Very significant statistical differences ($P<0.01^{**}$) were observed in the ramification length of the treatment groups P987Y and V13 when compared to the VEFG group.

Supplementary Table 1: VNAR V13 conformations by MD

Conformation		Number of structures	Existence time (ns)
1	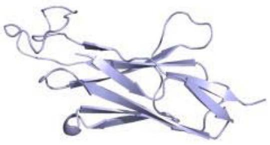	158	3.2
2	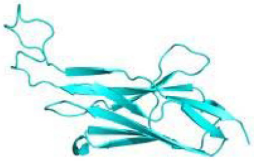	1821	36.3
3	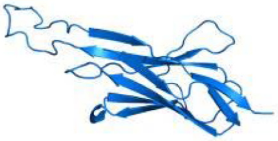	203	4.0
4	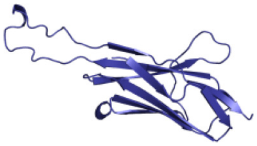	2074	41.4
5	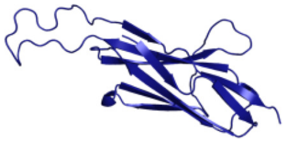	756	15.1
